# Thermographic Evaluation of the Duration of Skin Cooling After Cryotherapy in Dogs Following Tibial Plateau Leveling Osteotomy Surgery

**DOI:** 10.3389/fvets.2022.784327

**Published:** 2022-03-31

**Authors:** Sang Chul Woo, Jack Lee, Darryl L. Millis, Marti G. Drum

**Affiliations:** Department of Small Animal Clinical Sciences, University of Tennessee, Knoxville, Knoxville, TN, United States

**Keywords:** cryotherapy, thermography, physical therapy, TPLO, rehabilitation

## Abstract

**Objective:**

To evaluate the duration of cooling after cryotherapy on the skin over stifle joints in dogs after tibial plateau leveling osteotomy (TPLO) surgery using thermography.

**Materials and Methods:**

Seventeen client-owned dogs of various breeds were enrolled in the study. Dogs underwent TPLO surgeries, and on the next day, thermal imaging was performed on the operated stifle prior to cryotherapy for baseline. Orthogonal views were repeated at 30-min intervals until the skin over the stifle had thermally equilibrated. An ice pack was applied for 20 min on the medial and lateral aspects of the stifle. Each stifle was then re-imaged every 15 min for the first 60 min then every 30 min subsequently until the temperature was within 1°C of the pre-cryotherapy temperature.

**Results:**

Mean skin temperature of the medial view showed no significance difference compared to baseline value at 45 min after cryotherapy was discontinued and after 60 min for the lateral and cranial views. Mean skin temperature was overall higher in the medial view compared to the lateral and cranial during the rewarming period (except immediately after cold application). Mean skin temperatures of all views combined showed a significant decrease in temperature during cryotherapy application, with a slow increase until a plateau was reached after 45 min of rewarming.

**Conclusions and Clinical Relevance:**

Dogs undergoing TPLO for cranial cruciate ligament injury showed quicker rewarming period of superficial tissues compared to previous studies. Cryotherapy is a beneficial modality to reduce superficial tissue temperature in dogs undergoing TPLO, acknowledging that these dogs may require more frequent cryotherapy post-operatively due to more rapid rewarming time compared to dogs without surgery.

## Introduction

Cryotherapy, or the use of cold to cool tissues, is a commonly used rehabilitation modality following injury, surgery, and strenuous activity to relieve pain and minimize inflammation. In post-surgical patients, cryotherapy may be used in the immediate recovery period and following exercises in patients undergoing rehabilitation. There are several cryotherapy methods, including conduction (i.e., ice pack application with or without compression, cold whirlpools, ice massage, contrast baths, cold compression units), convection (immersion baths), and evaporation (vapocoolant sprays). The benefits of cryotherapy have been well documented, including, vasoconstriction, decreased cellular metabolism, decreased swelling, reduced enzyme-mediated tissue damage, and analgesia (1–7). Although some of these benefits persist after the affected area has rewarmed, analgesic effects due to decreased nerve conduction velocity persist only as long as the area remains cooled. The cutaneous thermal receptors that are responsible for temperature sensation response are functional between 25 and 36°C (8). The frequency of activation sharply decreases at temperatures below 20°C, and is minimal between 10 and 12°C.

Tibial Plateau Leveling Osteotomy (TPLO) is one of the most frequent procedures performed in dogs where post-operative cryotherapy is applied. The significant levels of tissue and bone damage induced by the surgery lead to edema, inflammation, and bruising around the stifle, making dogs undergoing TPLO good candidates for post-operative cryotherapy (9).

Temperature data from skin and deeper tissues has traditionally been collected *via* needle thermistors, which are invasive and require sedation or anesthesia in veterinary patients. Furthermore, medications used to induce sedation and anesthesia may cause significant hemodynamic changes in veterinary patients, including vasoconstriction or vasodilatation (10–12). These hemodynamic changes and possible decreased muscle contractions in the limb may alter rewarming times in clinical patients (13). In addition, needle thermistors only allow temperature data collection from a single region (14). Furthermore, young, healthy research dogs used in studies may not be reflective of the clinical patients with cranial cruciate ligament rupture that may include older, overweight animals (15–17).

Thermal imaging has been recognized as a potential diagnostic tool in human medicine by the American Medical Association council since 1987 and is being used with increasing frequency in human and veterinary medicine (18). Thermal imaging uses specialized infrared cameras that can detect radiation that has correlation with body surface temperature. Thermography use is based on this correlation of superficial temperature with various disease conditions (19). Studies have suggested thermography may be useful in the diagnosis of conditions such as human breast cancer, degenerative joint disease (20), sports injuries, and cardiac surgery (21), as well as equine lameness, canine intervertebral disc disease, and canine cranial cruciate ligament pathology (19, 22–24). Additionally, multiple studies have found it to be highly reproducible, with low inter-examiner variation (23, 25). Thermal imaging is advantageous as it is non-invasive and does not expose the patient or operator to radiation or the need for sedation or general anesthesia (26).

There are several previous studies that have used thermography to evaluate the effects of cryotherapy on skin temperature. A strong inverse relationship was reported between skin temperature and intramuscular temperature in one study using thermal imaging, inferring that intramuscular cooling occurs as skin temperature undergoes rewarming (27). Another study found no correlation between skin and intramuscular temperature during cooling but did find a relationship during rewarming (28).

To the authors' knowledge, this is the first study to use thermography to evaluate the effect of cryotherapy on the skin over the stifle in dogs post-TPLO surgeries. We hypothesized the skin temperature of the stifle in dogs that underwent TPLO surgeries would have a shorter rewarming period compared to those without surgeries.

## Materials and Methods

### Study Enrollment

Twenty client owned adult dogs undergoing tibial plateau leveling osteotomy (TPLO) surgery for cranial cruciate ligament rupture at the University of Tennessee College of Veterinary Medicine were evaluated between June 2016 to July 2017. The dogs were enrolled after obtaining written client consent and meeting the criteria of being otherwise healthy based on physical examination, orthopedic exam, radiographs, and pre-operative blood work. Dogs were not included if they had any medical history that would prevent them from undergoing anesthesia. Dogs were also not included if there were clinically apparent bilateral cruciate ligament injuries or the data collection was incomplete. All study procedures were reviewed and approved by the University of Tennessee Institutional Animal Care and Use Committee.

### Surgery

Dogs underwent routine anesthesia, surgical prepping and TPLO surgery. Surgeries were performed by two ACVS Diplomate surgeons (D.L.M. and J.P.W.). Orthogonal radiographs were obtained immediately after surgery to verify anatomic limb alignment and appropriate implant positioning. Post-operatively, dogs received appropriate analgesic treatment as determined by an anesthesiologist. A sterile adherent Telfa® island dressing (Covidien LP, Mansfield, MA, USA) was placed to cover the entire length of the incision (either 2 x 3″ or 3 x 4”), and a modified Robert Jones bandage was placed on the operated leg prior to continuing recovery in the intensive care unit.

### Thermography

**Figure 1:**
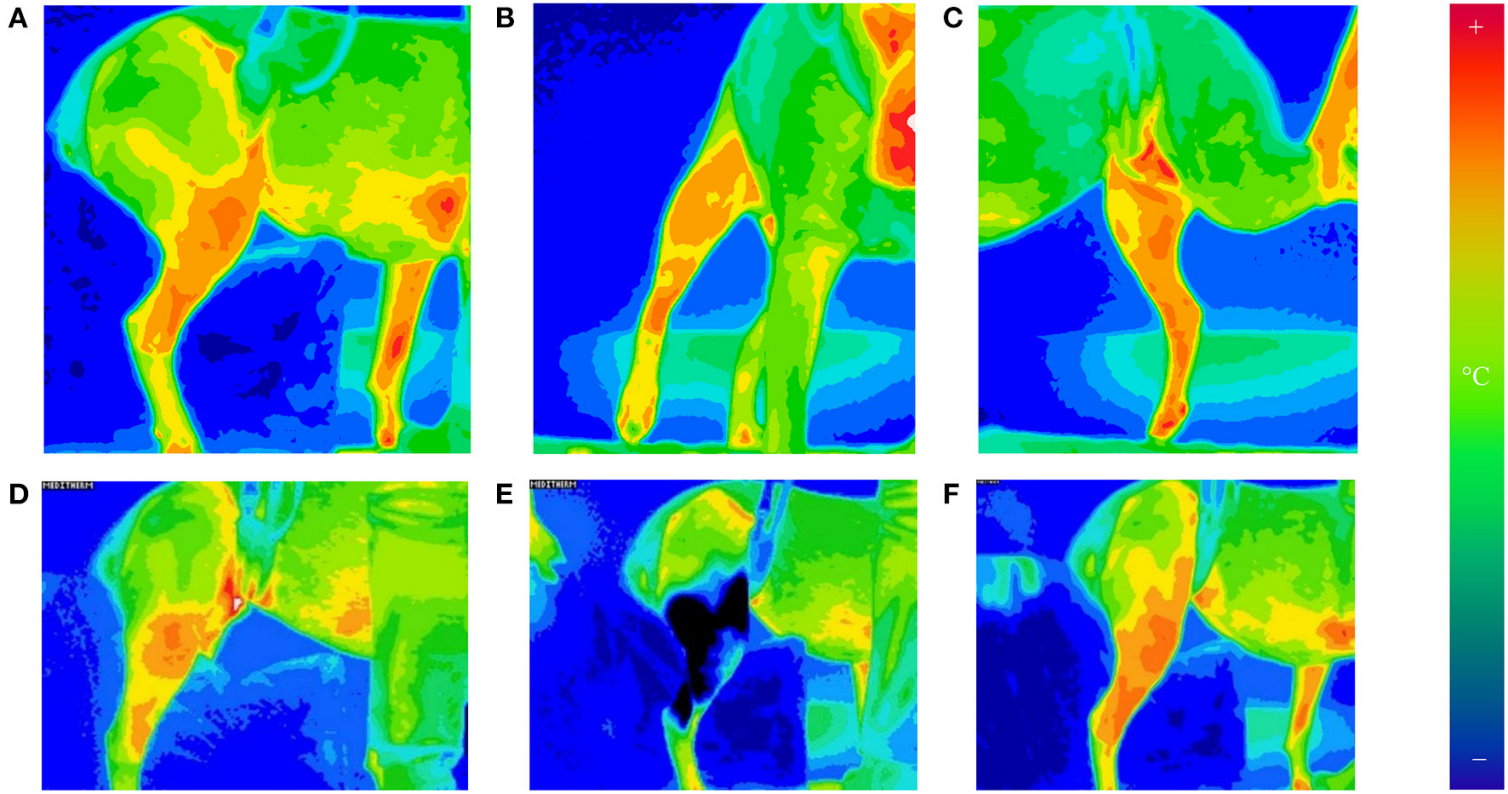
Thermographic images of orthogonal views of the operated stifle [lateral **(A)**, cranial **(B)**, and medial **(C)**]. Images taken of the lateral, cranial, and medial aspects of the stifle at 30-min intervals until equilibrated. The camera was set to a 16°C temperature scale and 16-shade color map, ranging from violet (coldest) to red (hottest). Thermography of lateral stifle at equilibrium is depicted on **(D)**. The stifles were equilibrated when two consecutive sets of images exhibit (30 min apart) similar heat distribution patterns globally. This was found to consistently occur by 1 h after bandage removal. After 20 min of cryotherapy, thermography of lateral stifle was obtained on **(E)**. Rewarming of the stifle is seen on **(F)**. Thermographic images of orthogonal views of the operated stifle © 2021 by Sang Chul Woo is licensed under CC BY 4.0. To view a copy of this license, visit http://creativecommons.org/licenses/by/4.0/.

The day after surgery, patients were relocated to a room with ambient temperature set of 21.0 ± 1.0°C for thermographic evaluation. The modified Robert Jones bandage was removed, but the Telfa® island dressing was left intact. Triplicates of thermographic images using a digital infrared thermal imaging system (Med2000 IRIS, Meditherm, Cheyenne, WY, USA) were obtained from operated stifles. The camera was centered at the stifle and measured instantaneous temperature at the given time. Orthogonal views (lateral, cranial, and medial) were obtained to determine mean temperature from a distance (between 50 and 150 cm) to include the entire hip and leg in the frame of the image with dogs in a standing position (Figures 1A–C). Thermal imaging was repeated at 30-min intervals until the stifle had thermally equilibrated after bandage removal. The stifle was considered as equilibrated when two consecutive sets of images exhibited similar heat distribution patterns within ±1°C in the stifle region. After equilibration was achieved, the Telfa® island dressing was removed, A supportive belly sling and a leash were used to minimize human-to-dog contact to prevent thermal artifacts. Between imaging acquisitions, dogs were placed on a padded blanket in lateral recumbency with the operated leg up.

After equilibration, ice compression was applied to the medial and lateral sides of the operated stifle for 20 min using clear plastic bags filled with crushed ice. Immediately following cryotherapy, thermographic images of the operated stifle were obtained in triplicate. Additional thermographic images were obtained every 15 min for the first hour after cryotherapy, followed by every 30 min until 2 h after cryotherapy. If mean temperatures of stifles remained below pre-cryotherapy levels at 2 h, data collection continued every 30 min until instantaneous temperature returned to within 1°C of pre-cryotherapy temperature for two consecutive time points (Figures 1D–F).

### Data Analysis

Analyses were conducted in SAS 9.4 TS1M7 (SAS Institute Inc., Cary, NC, USA). Differences in mean skin temperature between sides were analyzed using a repeated measures mixed effect model with group (side), time, and a side by time interaction. Due to statistical significance, the interaction term was kept in the model and individual differences between sides were subsequently evaluated using least squares means with Tukey's adjustment for multiple comparisons. Statistical significance was identified at the level of 0.05.

## Results

Twenty dogs underwent TPLO surgery without intra-operative complications. All but one dog tolerated the post-operative therapies. The dog was excluded from the study because of inability to maintain a calm, relaxed position during cryotherapy. Two other dogs were excluded due to incomplete data acquisition (*n* = 1) or inadvertent premature bandage removal (*n* = 1). Therefore, seventeen dogs underwent data analysis (Supplementary Table 1). One dog was used twice in the study, with TPLO performed 3 months apart (left followed by right TPLO).

There were 7 spayed females, 8 castrated males, and 2 intact males. The mean age of dogs was 6.27 ± 2.75 years, with a range of 2.5 to 10.5 years of age. Mean body condition score was 6.47 out of 9 ± 1.28. The average weight was 27.53 ± 10.28 kg, ranging from 13.2 to 45 kg. Breeds represented included Akita (*n* = 1), Australian Shepherd (*n* = 2), Boxer (*n* = 3), Brittany Spaniel (*n* = 1), German Shepherd (*n* = 1), Jack Russell Terrier (*n* = 1), Labrador Retriever (*n* = 2), Shih Tzu (*n* = 1), and mixed breed (*n* = 5). There were 10 left and 7 right TPLOs.

**Figure 2:**
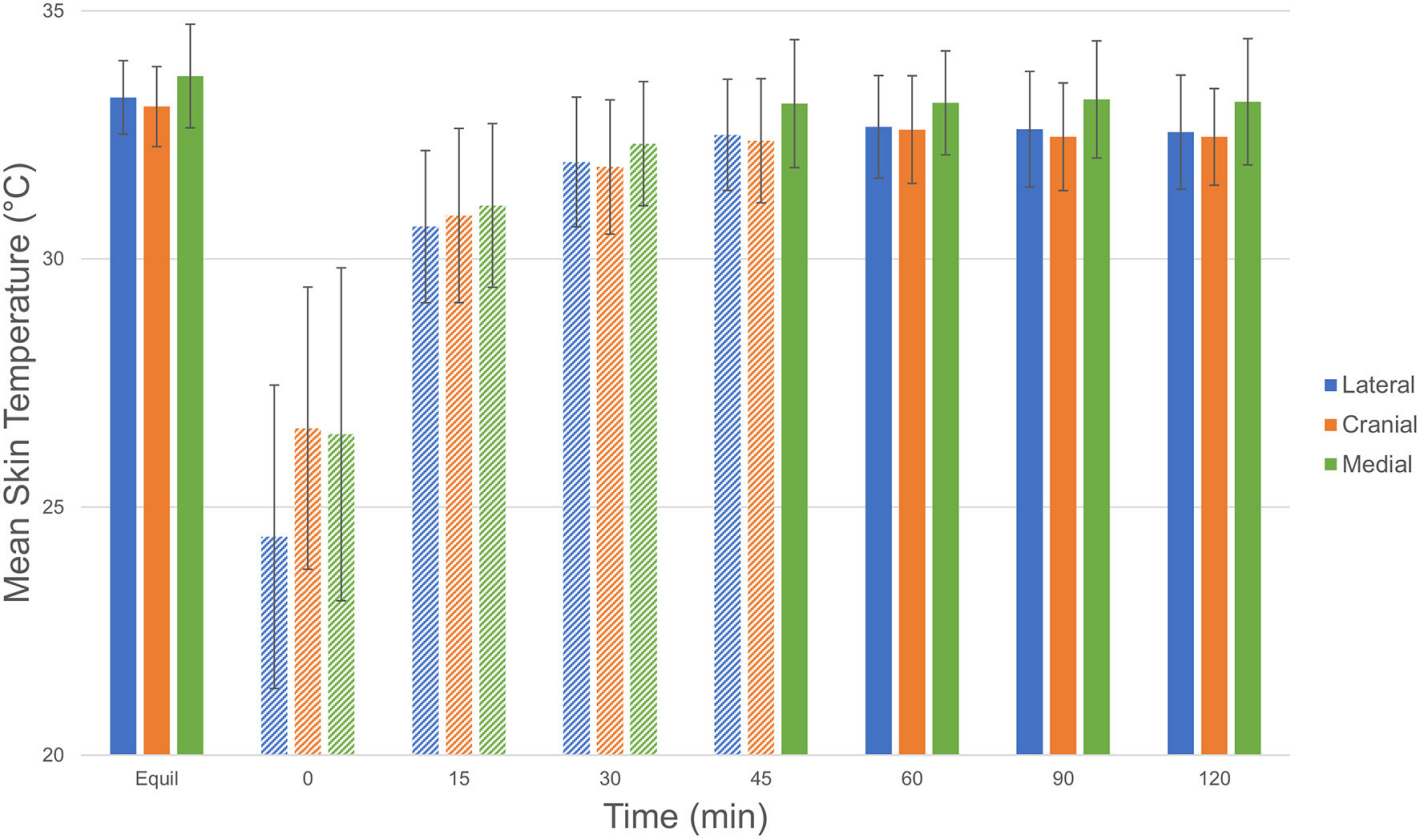
Comparison of mean ± SD (standard deviation) skin temperature (°C) of each view (lateral [blue], cranial [red], and medial [green]) at equilibrium, 0, 15, 30, 45, 60, 90, and 120 min after cryotherapy. Bars with striped pattern indicate those with significant difference (*p* < 0.001) from equilibrium. Comparison of mean ± SD (standard deviation) temperature (°C) of each view © 2021 by Sang Chul Woo is licensed under CC BY 4.0. To view a copy of this license, visit http://creativecommons.org/licenses/by/4.0/.

**Figure 3:**
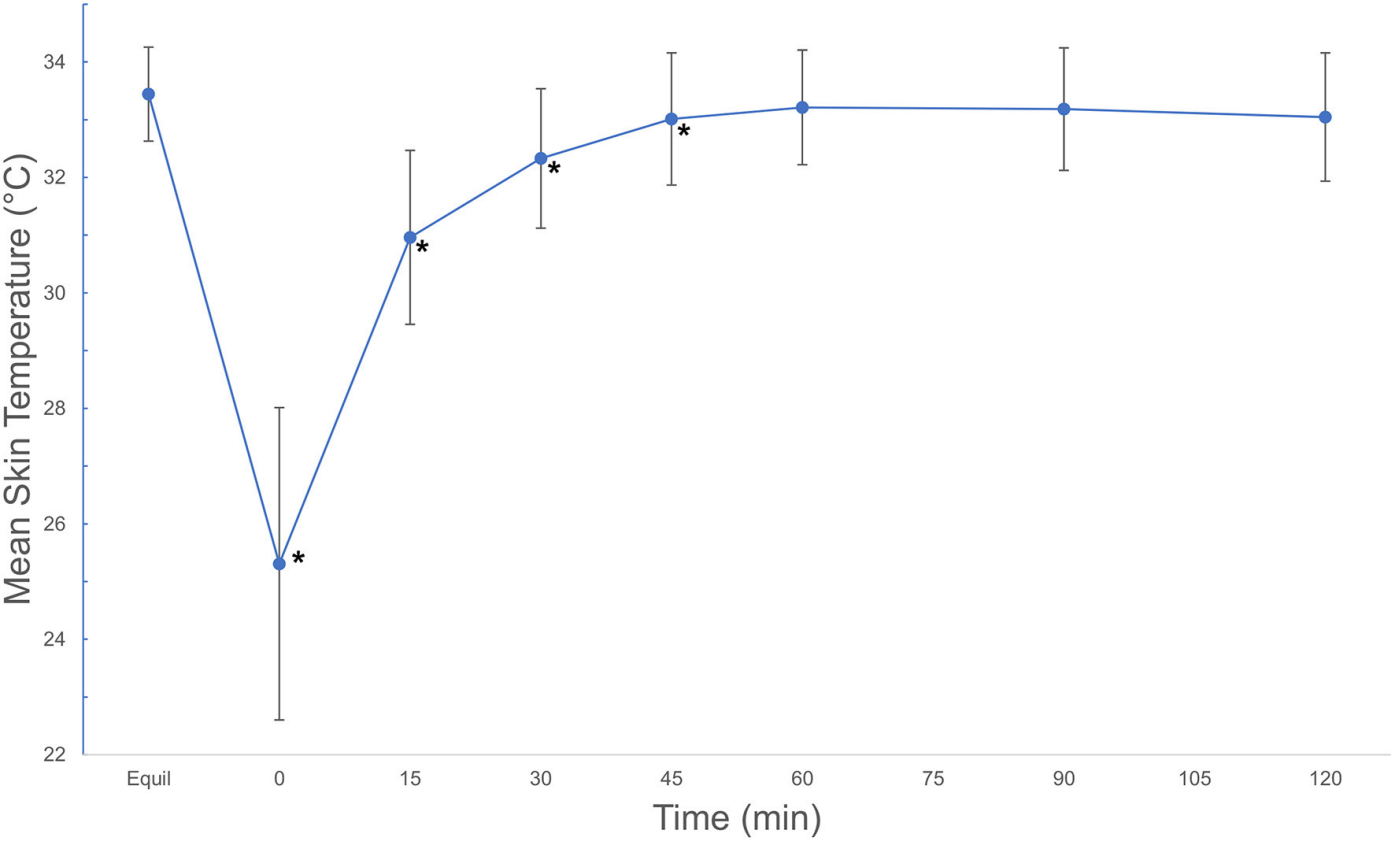
Mean ± SD skin temperatures (°C) of all views combined, beginning after an equilibration period, then continuing after a 20-min cryotherapy at 0 min and following rewarming for 120 min. Asterisks indicate significant difference from equilibrium (*p* < 0.001). Mean ± SD temperatures (°C) of all views combined, beginning after an equilibration period, then continuing after a 20-min cryotherapy at 0 min and following rewarming for 120 min. © 2021 by Sang Chul Woo is licensed under CC BY 4.0. To view a copy of this license, visit http://creativecommons.org/licenses/by/4.0/.

Mean skin temperature decreased by 7.4°C immediately after cryotherapy (0 min) and returned to baseline 45 mins after application on the medial aspect of the limb (Table 1). For the cranial and lateral aspects, the mean skin temperature decreased by 8.9 and 6.5°C, respectively, after cryotherapy (0 min) and returned to baseline 60 min after application for both aspects of the limb. Mean skin temperatures decreased significantly (*p* < 0.001) between equilibrium to immediately after cryotherapy on all views. Minimum temperatures reached immediately after cryotherapy were 20.8, 19.8, and 21.0°C for medial, lateral, cranial and views, respectively. At 15 min, the mean temperature elevation of all views significantly increased (*p* < 0.001). There was no significant temperature difference (*p* = 1.00) between the lateral and the cranial views; however, the mean temperature in the medial view was higher compared to the lateral and cranial views, except at 0 min where the cranial was the highest (Figure 2). Mean skin temperatures of all views combined showed a significant decrease of 7.6°C between equilibrium to 0 min, and then temperatures increased until they plateaued after 45 min (Figure 3). The mean temperature did not completely reach the pre-cryotherapy equilibrium temperature at 120 min, but by 60 min, the mean temperature was within 1°C of the equilibrium temperature.

**Table 1:** Mean ± SD skin temperature (°C) of each view at different times after cryotherapy application.

	**Mean** **±SD skin temperature (°C)**		
**Time (min)**	**Medial**	**Lateral**	**Cranial**
Equilibrium	33.7 ± 1.0	33.3 ± 0.7	33.1 ± 0.8
0	26.5 ± 3.4	24.4 ± 3.1	26.6 ± 2.8
15	31.1 ± 1.7	30.7 ± 1.5	30.9 ± 1.8
30	32.3 ± 1.3	31.9 ± 1.3	31.9 ± 1.4
45	33.1 ± 1.3	32.5 ± 1.1	32.4 ± 1.3
60	33.1 ± 1.0	32.7 ± 1.0	32.6 ± 1.1
90	33.2 ± 1.2	32.6 ± 1.2	32.5 ± 1.1
120	33.2 ± 1.3	32.6 ± 1.2	32.5 ± 1.0

## Discussion

The results of this study revealed that dogs undergoing TPLO for cranial cruciate ligament injury have a similar cooling and rewarming period after cryotherapy compared to previous studies (7). Mean surface temperature of the stifles a day after TPLO surgery no longer showed significant difference from pre-cryotherapy equilibrium temperature at 45 min for the medial aspect and at 60 min for the lateral and cranial aspects. When combined, the mean surface temperature showed no significant different from equilibrium at 45 min of rewarming period. We accepted our hypothesis of shorter rewarming period compared to previous studies evaluated on unoperated stifles, which reported a rewarming time of 60 min (4, 5, 7). This discrepancy between our findings and the previously reported rewarming time may be a result of acute inflammation and increased stifle joint temperatures following surgery.

The temperature in the medial view was overall higher than the lateral or cranial views. The temperature in the medial aspect also had faster rewarming than the other views. In a study by Infernuso et al. (22) using thermography of surface temperature over stifle in CCL-deficient dogs compared to uninjured stifles, the average equilibrium skin temperatures of a CCL-deficient stifles prior to surgery were 31.48, 31.15, and 31.85°C for lateral, cranial, and medial views, respectively. This was lower than the equilibrium temperatures found in our study of 33.26, 33.07, and 33.69°C for lateral, cranial, and medial views, respectively. Although it is difficult to draw firm conclusions in comparing the two studies due to variation in study design, it suggests that the increased temperature in our study can associated with inflammation following surgery. Studies have demonstrated that joint temperature increases when joint pathology exists (20). While the regulation of stifle temperature may not seem to be as important as other treatments after surgery, studies have demonstrated increased levels of prostaglandin E_2_ (PGE_2_) and cellular energy consumption with increased temperature in knees (29). Elevated concentrations of PGE_2_ have been associated with increased pain and the sensitization of peripheral nerves (29, 30). Additionally, increased energy consumption (leading to a hypermetabolic state) has been associated with increased lactate, tissue damage, and cell death. The differences in temperature on the medial aspect is greater, perhaps due to the location of the incision and underlying tissue dissection which result in greater inflammation and therefore, skin temperature.

Although the present study did not evaluate deeper tissues because of the nature of thermal imaging, the temperature changes in the superficial tissues are similar to those of studies that have used thermistors to measure temperature changes in superficial and deeper tissues (6, 7). Other studies have shown less decrease in deeper tissue temperature and more gradual rewarming of these tissues compared with superficial tissues (3, 7). This is likely due to the second law of thermodynamics which states that heat is transferred from an area of warmer temperature to an area of cooler temperature. This may explain the rapid cooling of superficial tissues as heat is transferred from the tissues to the ice pack, and the rapid rewarming of superficial tissues after cryotherapy as heat moves from deep tissues to superficial tissues. Similarly, as heat moves from the deeper tissues to warm superficial tissues, the deeper tissues may have more prolonged tissue cooling, although the effect is less dramatic than superficial tissue temperature changes.

Although we did not measure the temperature of deeper tissues, the temperature changes in the superficial tissues measured in this study are similar to those using thermistor measurements of tissue temperature, and it is logical to believe that changes in temperature of deeper tissues may be similar to deep tissue temperature changes found in other studies. Because of the similarity of superficial tissue temperature changes in these studies, it may be possible to use thermal imaging to predict when cryotherapy may need to be reapplied to maintain therapeutic cooling. This is advantageous because thermal imaging is non-invasive compared to other techniques of tissue temperature measurement. However, further study of the effect of post-surgical cryotherapy on deeper tissues and the use of thermal imaging to predict these changes is warranted.

One limitation of the present study was the method of temperature measurement. To the authors' knowledge, there are no previous studies using thermography to evaluate cryotherapy on patients undergoing TPLO surgery; therefore, there is lack of data for comparison. The study by Infernuso et al. (22) obtained measurements in a room with ambient temperatures between 20 to 24°C, which was more variable than the room temperature in our study. In addition, we evaluated dogs after surgery while their study evaluated dogs prior to surgery. A study by Janas et al. (7), examined surface temperatures of normal clipped thighs after 20 min of cryotherapy using thermistors. The stifles of clinically affected dogs in this study exhibited higher mean surface temperature at 25.8°C compared to 18.2°C from the skin of the thigh after 20 min of cryotherapy. The differences may be due to the manner of temperature acquisition (thermistor vs. thermal imaging camera), sites of data acquisition (thigh vs. stifle), anesthetized vs. awake dogs, or pathology (normal dogs vs. those undergoing TPLO surgery). The variation in distance to limb during thermography can also impact the mean surface temperature and standard deviation within 20 m but may be less significant in distances used in this study (31). This variation may report to under-estimation of surface temperature. Because of the variety of patients and experimental models, instrumentation, and method of cryotherapy, direct comparison of results can be challenging. While the trends are similar among studies, it is difficult to make definitive quantitative comparisons.

Another limitation of the present study was the number of dogs evaluated, and variability in the body condition score (BCS) and the body size of dogs. The dogs in this study had varying BCS, from 5 out of 9 to 9 out of 9. There was no overall trend of temperature changes among dogs with different BCS. Due to higher adipose in dogs with higher BCS, there were concerns that dogs with higher BCS may have greater cooling effect due to low thermal conductivity and thermal diffusivity of adipose tissues (6). However, this was not evident in this study. However, the impact of body condition scores on thermographic measurements warrants further investigation.

Future work should be performed to evaluate the effect of cryotherapy on deeper tissue temperatures after TPLO. In addition, the method of cryotherapy following surgery should be further evaluated. Although cold compression is an easily accessible method of cryotherapy, it has also been shown to be among the more short-lived in duration. Full immersion and compressive cooling sleeves result in greater tissue temperature reduction and longer duration of action. However, if the inflammation and edema that result from surgical trauma are the cause of the more rapid rewarming, this may also occur with these other modalities. Further study of non-invasive thermal imaging is warranted to better understand the effect of cryotherapy in the clinical setting.

We concluded that cryotherapy is a beneficial mode to reducing superficial tissue temperature in dogs undergoing TPLO but also acknowledge that these dogs may require more frequent cryotherapy post-operatively due to more rapid rewarming time compared to unoperated dogs. The results also support the belief that the medial aspect may be warmer due to inflammation from the incision and surgical site, and differences in the composition of tissues under the skin.

## Data Availability Statement

The original contributions presented in the study are included in the article/Supplementary Material, further inquiries can be directed to the corresponding author/s.

## Ethics Statement

The animal study was reviewed and approved by University of Tennessee Institutional Animal Care and Use Committee. Written informed consent was obtained from the owners for the participation of their animals in this study.

## Author Contributions

MD and DM conceptualized the study design. JL, MD, and DM contributed to the acquisition of data. SW and DM analyzed and interpreted the data. All authors publicly accountable for relevant content, drafted and revised, and approved submitted manuscript.

## Funding

This study was funded by the University of Tennessee Small Animal Canine Arthritis Rehabilitation Exercise & Sports Medicine (CARES).

## Conflict of Interest

The authors declare that the research was conducted in the absence of any commercial or financial relationships that could be construed as a potential conflict of interest.

## Publisher's Note

All claims expressed in this article are solely those of the authors and do not necessarily represent those of their affiliated organizations, or those of the publisher, the editors and the reviewers. Any product that may be evaluated in this article, or claim that may be made by its manufacturer, is not guaranteed or endorsed by the publisher.

## Abbreviations

BCS: Body condition score
CCL: Cranial cruciate ligament
TPLO: Tibial plateau leveling osteotomy
SD: Standard deviation.
