# Supplementary material for: Thermographic Evaluation of the Duration of Skin Cooling After Cryotherapy in Dogs Following Tibial Plateau Leveling Osteotomy Surgery
Source: Front Vet Sci. 2022 Mar 31;9:784327. doi: 10.3389/fvets.2022.784327 (PMC9009085; doi:10.3389/fvets.2022.784327)
Supplement: Supplementary file 1 [file Table_1.pdf]

Supplementary Table 1. Case details of 17 dogs.

Case No.	Breed	Sex	Age (yr)	Weight (kg)	BCS (/9)
1	Mixed	MC	2.50	36.5	6
2	Akita	FS	6.48	38	6
3	Boxer	MC	2.48	38	7
4	Mixed	MC	7.50	38	8
5	Australian Shepherd	FS	6.66	20	8
6	Boxer	FS	6.55	26	5
7	German Shepherd	FS	3.57	31.7	5
8	Mixed	MC	10.49	18.55	7
9	Labrador Retriever	FS	9.56	31	5
10	Labrador Retriever	MI	3.59	45	8
11	Australian Shepherd	FS	6.93	19.2	7
12	Mixed	FS	9.96	16.4	5
13	Mixed	MC	4.95	27.5	6
14	Boxer	MC	5.40	38	6
15	Jack Russell Terrier	MI	9.41	13.2	7
16	Brittany Spaniel	MC	2.18	17.3	5
17	Shih Tzu	MC	8.38	13.6	9

**Surgery side**
R
L
L
R
L
L
R
L
R
R
R
L
L
L
R
L
L
